# Institutional Notes and News

**Published:** 1910-05-14

**Authors:** 


### Institutional Notes and News.

**Bedford County Hospital.**

Lord Ampthill, Chairman of the Bedford County Hospital, in remarking at the recent annual meeting on the little interest that was taken by the general body of governors in the election of the Board of Management, made a speech which deserves to go beyond the audience to which it was immediately addressed. He is reported as follows: "I rather wish there was a little more interest in the matter of representatives among the general body of governors. We do not want contested elections, but we never get even a suggestion from the general body; they seem to have such confidence that they leave it to us to nominate one another, and if we did not do it no one would be nominated, and it would be impossible to elect anyone, as under the rules 21 days' nomination are required. But although they seem to have such implicit confidence in us, there is a good deal of complaint. I have heard, for instance, a complaint that the tradesmen have no representative. There need be none, because any subscriber can nominate one. The Board, two years ago, thought it might be more representative, and did their best to make it so. They approached 12 or 14 tradesmen, but one and all said they could not afford the time. That being so, I do not think it lay with them to say they were not represented. Still, I wish those who complained would come and serve themselves or get their friends to do so. We should be delighted to welcome any representative of the tradesmen; they are very good to the Hospital, and if they desired representation they should have it." The daily average number of patients for the year was 71.86; in-patients numbered 722, out-patients 3,370. As the year began with a debt of £500, the figures for this year's accounts—income £5,360, and expenditure £4,965—are quite satisfactory.

**Children's Hospital, Nottingham.**

The Nottingham Children's Hospital can congratulate itself on having practically escaped from the financial difficulties which a year ago were represented by a debt of £2,000 to its bank. The undignified and often unsatisfactory device of a bazaar was rejected, and the board took the businesslike course of issuing an appeal. So well was this answered that nearly £1,800 was subscribed, which has been credited to the capital account. Admissions to the wards for the year just ended numbered 492, and 6,819 cases were treated in the out-patient department. The total income amounted to £2,564, and the total expenses were £2,867, leaving a debt on the year's work of £303, to remind all the hospital's supporters that the constant war between credit and debit is only won by a steady stream of work. Sporadic bursts of energy and the forlorn hopes of bazaars only postpone the day of reckoning. We are sorry to observe the retirement of the Rev. H. C. Russell, who for more than a generation has been chairman of the board of management. This office is no light task, and, even if it were, twenty-eight years of it are enough to prove that its holder's enthusiasm has been a constant and living one, which must have carried him and the hospital through many a crisis in its course.

At the recent quarterly Court of Governors of the North Devon Infirmary an appeal for funds was issued, and Mr. C. E. R. Chanter, the chairman, announced that up to the present the House Committee had been compelled to close one of the wards. This has roused one section of the public at least, and this month two performances of the "Duke of Killiecrankie" are to be given in aid of the hospital by some ladies and gentlemen of Bideford. A debt of £500 is expected for the ensuing quarter, and if donations are not forthcoming a sale of invested stock is feared. By the closing of the ward already mentioned the number of beds has been cut down from sixty to forty, but as five of these are kept for possible emergencies, it means in practice that only thirty-five beds are now available. The dental department of the hospital, which was started nominally a little while ago, is being inquired into, so that the attendances of the honorary dentists may coincide with the hitherto somewhat uncertain calls upon their time. Legacies are badly wanted.

**North Devon Infirmary.**

After twenty years of work under the well-known name of the Manchester Eye and Ear Hospital, in St. John Street, the institution has been re-christened. It is believed that the new name, St. John's Hospital of Manchester and Salford for the Ear and Eye, will bind the hospital closer to the public spirit of Salford, from which, Dr. Westmacott remarked at the annual meeting the other day, he thought almost a third of the patients came. The Rev. J. G. Skemp, the honorary secretary, gave the number of in-patients for the year as 561, while the total attendances were 16,374. Dr. Renshaw pointed out that no fewer than seventy medical men of the district had sent patients to the hospital, and by an analysis of figures showed that 252 consultations were given in the out-patient department for every sovereign subscribed. The statement of accounts was presented by Mr. J. T. Lingard, the honorary treasurer, and he had to record a debt of £182 for the year, the income being £732, and the expenditure £914. It should be added that the patients of both departments were responsible for £588 of the total sum received, including their payments for medicines.

**St. John's Hospital, Manchester and Salford.**

The Malvern Hospital, the main object of which is to have major operations performed locally, and so to avoid the necessity of a journey to big hospitals at Worcester and elsewhere, has presented a satisfactory report to its annual subscribers' meeting. The patients treated numbered 401, an increase of eighty-six on the previous year's figures, while there is a balance on the right side of £4 10s., and this in spite of the fact that the year began with a deficit. This deficit was the serious one of £76, and if we add to it the balance of £4 we get the fine total of £80, for which the Hospital Saturday Collection was responsible, and which therefore deserves great credit for pulling the hospital out of a difficulty. Mr. and Mrs. Henry Urwick, however, contributed a very useful £50 by the success of their entertainment, without which and the President's (Sir Henry Foley Grey) contribution of the rent paid for the use of his common by the roundabouts, the great effort of the Saturday Fund would have been hampered seriously. The Ladies' Linen Guild has proved itself here again an indispensable institution, and the Malvern Hospital must be added to the list of those which, having now started it, are proofs of its value to others who have not done so yet.

**Malvern Hospital.**

The need for further accommodation for male patients at the Thames Ditton Cottage Hospital has led the Committee to acquire the option of leasing an adjoining site, where a new ward will be built as soon as the money is found. The amount needed for this is expected to be £750, for which £147 is now in reserve. The Shilling Fund, which was started by a sub-committee of ladies to raise the balance, has realised £580, so the remaining sum needed is very small. During the past year 117 patients have been treated. After the expenditure had been met, there remained a balance in hand of roughly £47, so, though we understand this is smaller than in the previous year, the hospital can congratulate itself on its financial position. Another cause of satisfaction has been provided by the gracious consent of H.R.H. the Duchess of Albany to become a patron, and the new ward when built will be christened, by her permission, the Albany Ward.

**Cottage Hospital, Thames Ditton.**

The reconstruction of the Chesterfield and North Derbyshire Hospital, begun two years ago, was completed last month. Some idea of its extent may be gained from the fact that the accommodation has been increased from eighty beds to 120; the nurses' quarters of the original hospital have been converted into wards, a new nurses' home has been built, and extensive renovation has been carried out in the basement offices. The new surgery, with the anæsthetist's room attached to the operating theatre, came in for special praise from Dr. Court, who, with other medical men, has visited the hospital on the invitation of the Board of Management. The chairman of the board, Mr. E. C. Barnes, has had a very busy time, and it is largely due to his energy and enthusiasm that these changes, at a cost of £20,000, of which £11,200 has been raised, have been carried through successfully. The new children's ward, the installation of electric light, the lift, and the hot-water and radiator systems have given in fact to Chesterfield what Dr. Court described as "one of the most modern hospitals in the Midland counties."

**The new Chesterfield and North Derbyshire.**

The Governors of the Meath Hospital and County Dublin Infirmary were confronted with serious want of money at their recent annual meeting. The year which they were met to consider began with an accumulated debt of £5,227, and increased it during its course by, roughly, £600 more. The hospital now owes £5,825. The annual report consequently was at pains to show that this was no fault of the management, and an important place is given to repeating the eulogies which the Hospitals Committee of the Dublin Corporation and the Hospitals Board of Superintendence made after paying surprise visits. The explanation then follows, and amounts to this, that if no patients who come for admission are turned away, while no fresh support is forthcoming, the hospital's debt is bound to grow. The figures are : total ordinary income £6,180, total expenditure £6,778. It is fair to add that some £400 was spent on drainage and £284 on painting in the course of the year. These expenses were met out of capital. The number of in-patients treated was 1,541, and the daily average number of occupied beds was 115. Accident and dispensary cases totalled 18,202. Sir Lambert Ormsby made a vigorous appeal for money at the annual meeting, pointing out that the hospital could look back on 156 years of work, and, being constituted by Act of Parliament, had a claim upon the rich householders of County Dublin, apart from the fact that it often treated their servants and employés free of charge.

**Meath Hospital.**

